# Lesions caused by Africanized honeybee stings in three cattle in Brazil

**DOI:** 10.1186/1678-9199-19-18

**Published:** 2013-08-22

**Authors:** Saulo Andrade Caldas, Flávio Augusto Soares Graça, Júlia Soares Monteiro de Barros, Márcia Farias Rolim, Tiago da Cunha Peixoto, Paulo Vargas Peixoto

**Affiliations:** 1 Department of Veterinary Surgery and Medicine, Institute of Veterinary Medicine, Federal Rural University of Rio de Janeiro (UFRRJ), Seropédica, Rio de Janeiro, Brazil; 2 Center of Agricultural Sciences and Technology, State University of Norte Fluminense, Rio de Janeiro, Rio de Janeiro, Brazil; 3 Undergraduate Program in Veterinary Medicine, Castelo Branco University, Rio de Janeiro, Rio de Janeiro, Brazil; 4 Graduate Program in Veterinary Medicine, State University of Norte Fluminense, Rio de Janeiro, Rio de Janeiro, Brazil; 5 Department of Pathology and Clinical Medicine, School of Veterinary Medicine, Federal University of Bahia (UFBA), Salvador, Bahia, Brazil; 6 Department of Animal Nutrition and Pastures, Institute of Animal Husbrandry, Federal Rural University of Rio de Janeiro (UFRRJ), Seropédica, Rio de Janeiro, Brazil

**Keywords:** Cattle, Bees, Sting, Poisoning, Accidents

## Abstract

We report three cases of stings by Africanized bees in cattle in the state of Rio de Janeiro, Brazil. Erythema, subcutaneous edema, necrosis accompanied by skin detachment, and subsequent skin regeneration were observed, especially on the head and dewlap. Histopathological examinations performed 45 days later revealed complete skin reepithelialization with moderate dermal fibrosis. The clinical picture and differential diagnosis are discussed in the present manuscript, with a focus on photosensitization, which causes cutaneous lesions on the head (sequela) with cicatricial curving of the ears and can be very similar to what is observed in cattle attacked by swarms of bees. The distinction between photosensitization and bee sting lesions can be made with a focus on history and clinical and pathological aspects.

## Background

The literature presents only a few substantiated reports regarding the clinicopathological picture of domestic animals stung by insects; the most frequent and most severe cases occur when Africanized bees collectively attack a single target [1].

Africanized bees, which were derived from the crossbreeding of European honey bees (*Apis mellifera* and *Apis lingustica*) and African honey bees (*Apis mellifera scutellata*), were introduced in Brazil in 1956 with the aim of increasing honey production [2]; however, they exhibit intense defensive behavior and frequently attack humans and animals [3,4]. The proportion and toxicity of the bees’ venom components can vary according to the season, the age of the bees, and even the flowers used by the bees for honey production [5].

The basic composition of the venom, although it has been poorly characterized biochemically, includes a mixture of enzymes, low molecular weight polycationic peptides, biogenic amines, and proteins of high allergenic potential [6-9]. The primary components of the venom include melittin and phospholipase A2, which represent 50 to 75% of the total venom mass [5,9]. Apamin, a peptide that causes changes in neurotransmission, comprises only 2% of the venom dry weight and affects the central and peripheral nervous system by blocking the transmission of some inhibitory impulses [10,11]. Honeybee venom also contains a mast cell degranulating peptide that is responsible for the release of histamine, serotonin, arachidonic acid derivatives, and some factors that act on platelets and eosinophils [5,11,12]. Histamine and hyaluronidase in the venom are responsible for the diffusion of the poison within the victim by decreasing blood pressure and increasing vascular permeability [13].

Melittin, a protein fraction that causes pain and inflammation, is responsible for the overall toxic effect of the venom by preventing the action of cholinesterase at neuromuscular and ganglionic synapses, causing respiratory paralysis and fibrinogen coagulation [13,14]. Melittin contains the amino acids leucine, glycine, alanine, isoleucine, threonine, lysine, arginine, and glutamic acid. The synergistic action of melittin with phospholipase A2 on phospholipids impairs cell and mitochondrial membrane integrity, alters oxidative phosphorylation, and causes tissue damage [11]. Melittin causes the release of lecithin from red blood cells, which is transformed into lysolecithin through the action of phospholipase A2, causing hemolysis [14].

Phospholipase A2 also acts on tissue respiration and prevents the action of dehydrogenases, in addition to inactivating thromboplastin [13]. Furthermore, this enzyme induces the release of prostaglandins that modify vascular permeability, which can even result in anaphylaxis [15,16].

The allergenic factors in bee venom consist of hyaluronidases that are responsible for the hydrolysis of hyaluronic acid, in addition to lipases and phosphatases that act on the lysis processes that occur in different tissues and increase the severity of the injury [5]. The hydrolytic actions of these enzymes are complemented by the actions of other enzymes (lipase, phosphate, and esterase) [6]. Additionally, the venom contains cardioprep, a non-toxic peptide that has a similar mode of action to that of beta-adrenergic drugs and has antiarrhythmic properties [5].

Although the occasional occurrence of bee stings in cattle in the fields has been verbally reported by veterinarians, owners, and handlers, we have failed to find clinicopathological descriptions of bee stings in cattle. To our knowledge, accidents involving bee stings in animals have only been described in detail in dogs.

In Brazil, severe systemic reactions have been reported in 19 dogs stung and killed by numerous Africanized bees between 1996 and 2006; these animals presented with congested mucous membranes, dyspnea, vomiting, red urine, bloody stools (hematochezia), laryngeal edema and epistaxis, central nervous system depression, shock, bleeding, liver and kidney damage, hypoproteinemia, and disseminated intravascular coagulation [11,17,18].

The aim of the present study was to describe the occurrence of non-fatal poisoning in three cattle attacked by Africanized bees, contribute data to the design of the clinicopathological status when surviving numerous stings and to establish the differential diagnosis for cases of photosensitization sequelae in cattle due to the high similarity between these lesions and bee stings.

## Case presentation

The first episode of bee stings in cattle that we report in the present manuscript occurred in July of 2010, in the city of Valença, Rio de Janeiro State, Brazil. A 5-year-old crossbred cow (cow 1), weighing 450 kg of live weight, was attacked by a large number of Africanized bees when it approached a beehive that was established in an inactive termite mound. One year later, again in the month of July, a 4-year-old crossbred cow (cow 2), weighing 460 kg, and a 6-month-old calf, weighing 130 kg, that were kept in the same pasture were attacked by a bee swarm when they approached the same termite mound.

**Figure 1:**
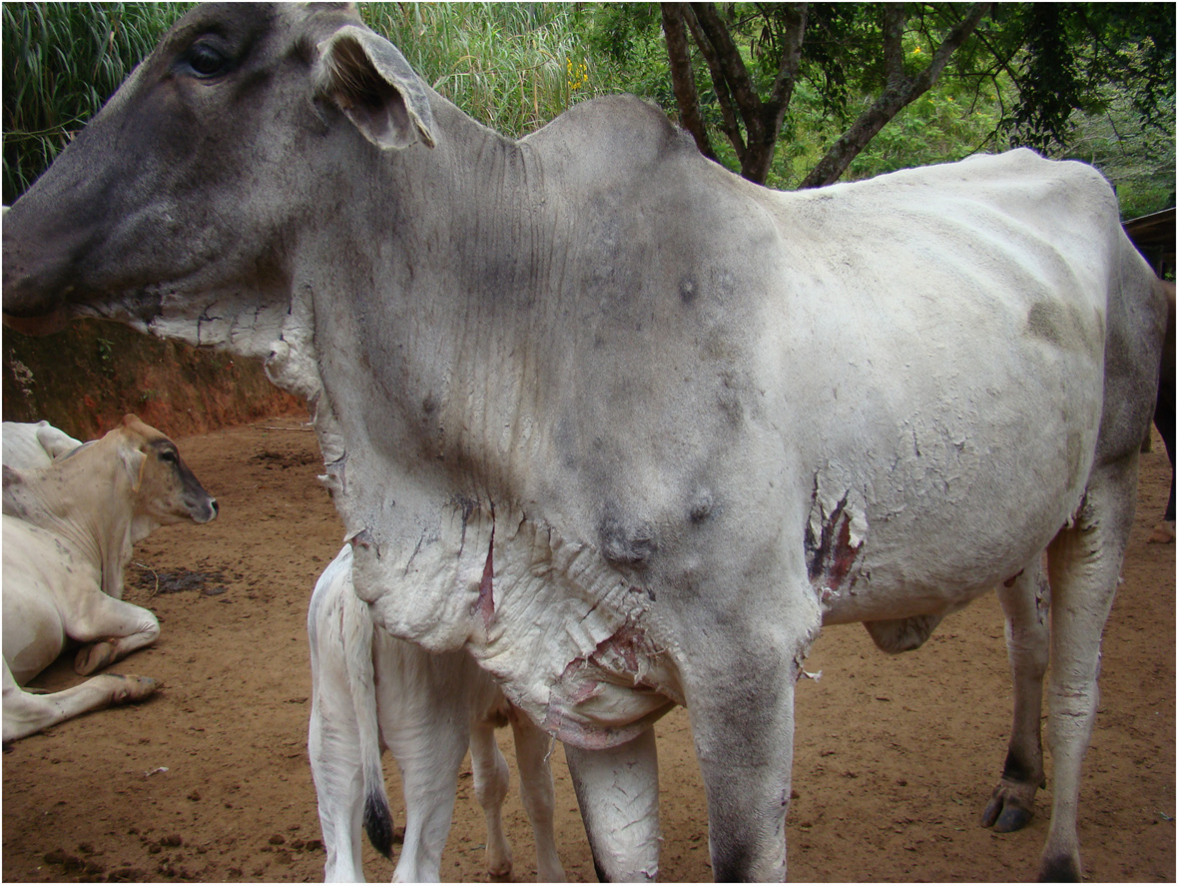
**Crossbred cow 17 days after the bee swarm attack.** Observe the necrosis and detachment of the skin on the dewlap and lower portion of the chest.

**Figure 2:**
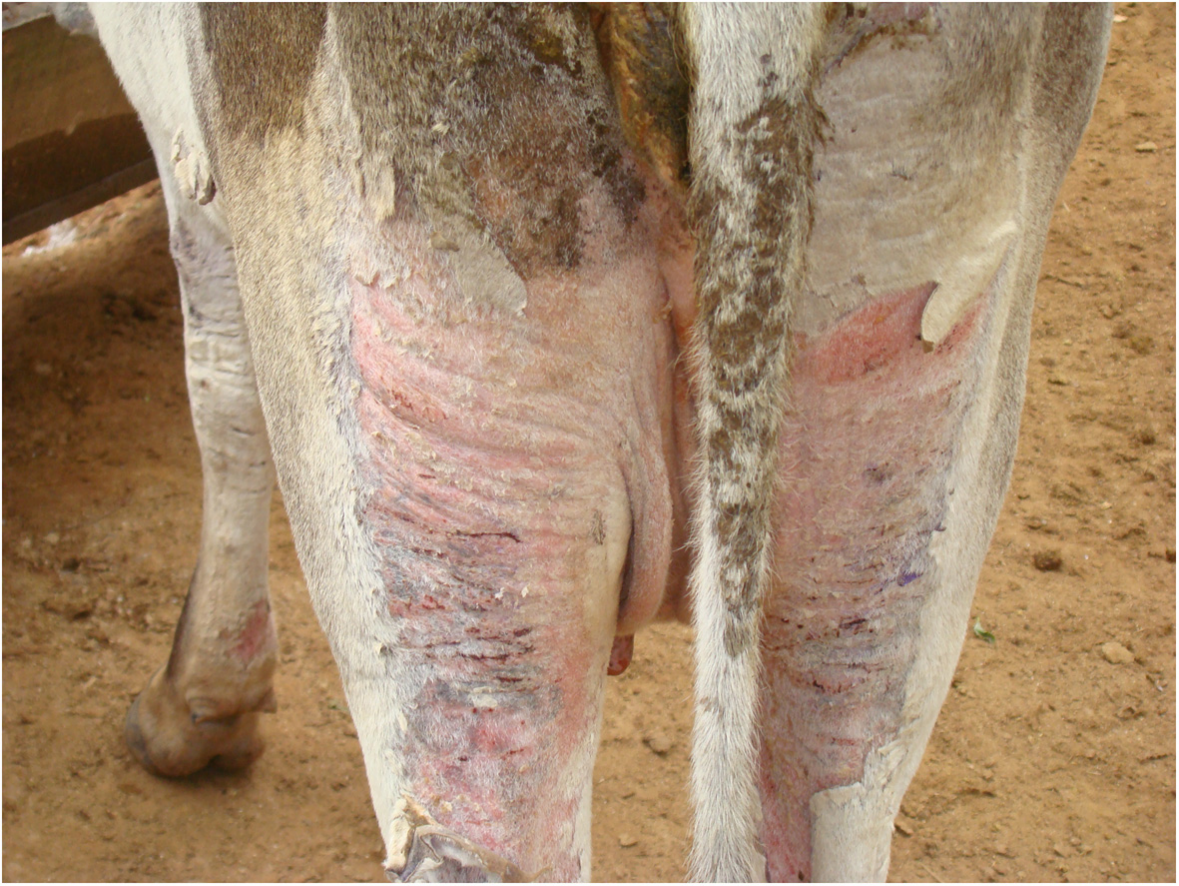
**Crossbred cow 17 days after the attack swarm of bees.** Observe the necrosis and detachment of skin on the hind limbs.

**Figure 3:**
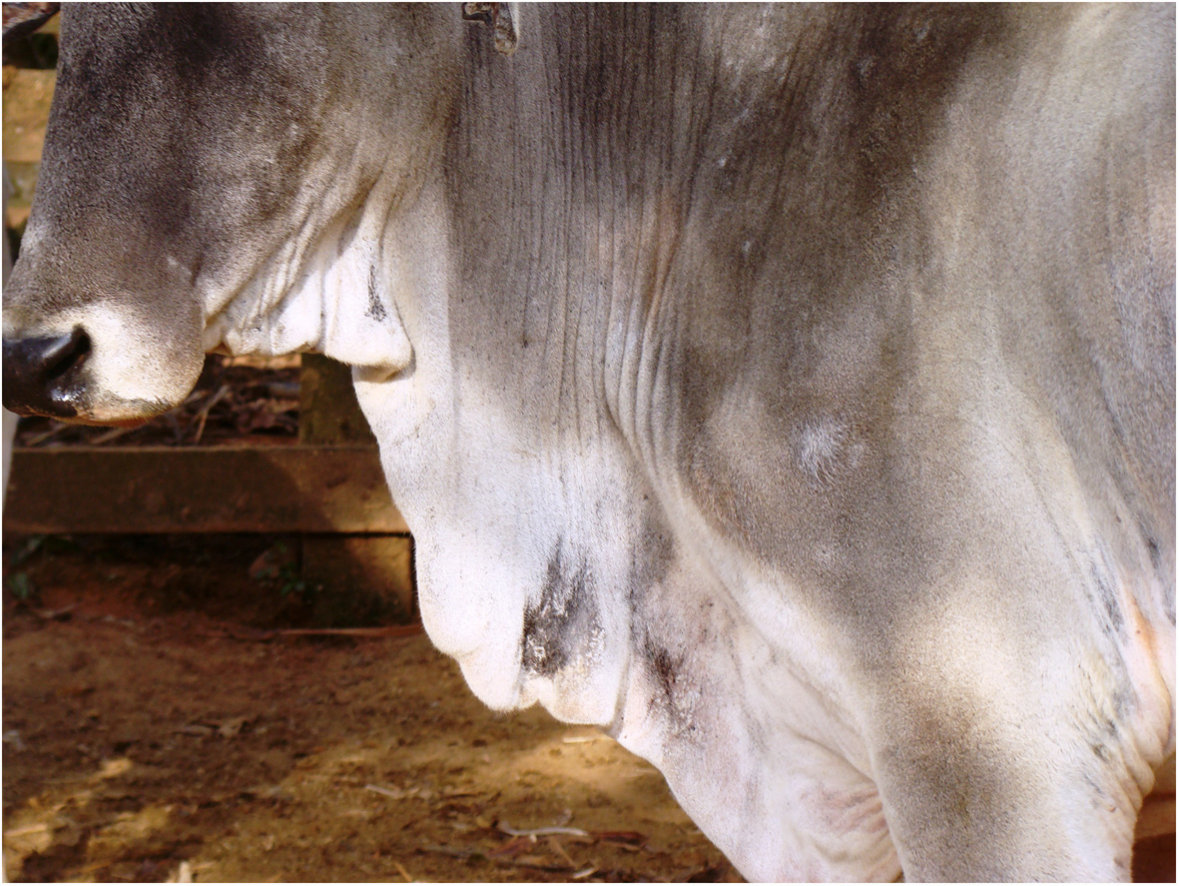
**Crossbred cow 45 days after the bee swarm attack.** Observe the healed lesions on the dewlap and lower portion of the chest.

**Figure 4:**
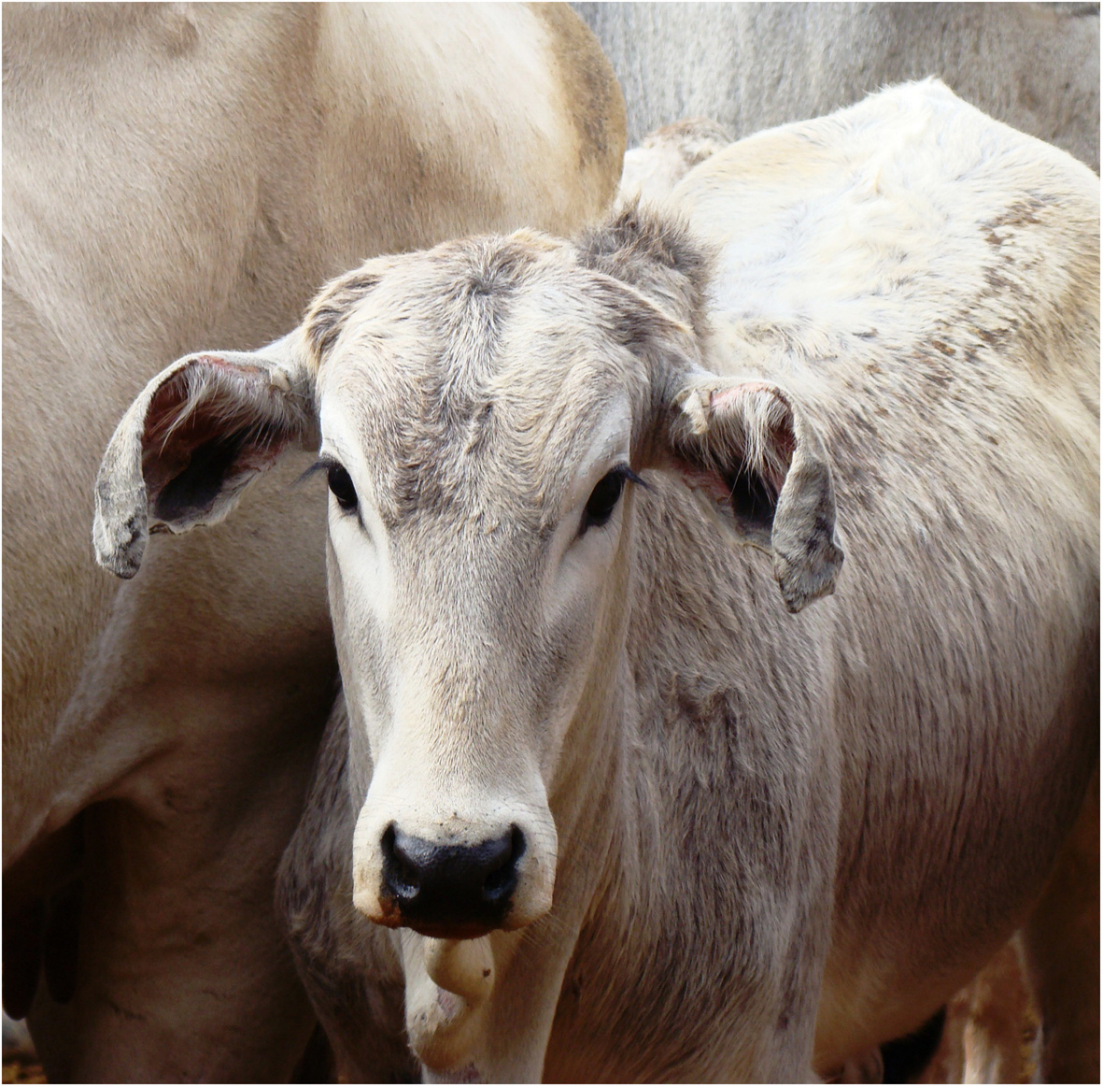
Cicatricial retraction of the ear, popularly known in Brazil as “cartridge-shaped ears” (calf).

Each of the attacked cows had 200 to 300 stingers on the skin, especially on the ears, dewlap, sternal region, abdominal-ventral regions, and back of the thigh. In all three animals, the most affected areas corresponded to the locations where more stingers were found. Within the first 48 hours after the attacks, the animals exhibited apathy, erythema at the sting sites, and marked swelling (edema with positive Godet sign), especially on the subcutaneous tissue of the head, dewlap, and caudal aspect of the hind limbs. Seven days after the attack, necrosis was observed at these same sites; later, detachment of the skin took place (Figures 1 and 2). Skin reepithelialization (Figure 3) and the cicatricial retraction of the ears (Figure 4) occurred within 45 days following the accidents. An excisional biopsy of the dewlap, which exhibited scarring and local alopecia, was performed in one of the animals (cow 2) 1.5 months following the attack, for histopathological evaluation. The histopathological examination revealed the complete reepithelialization of the skin, with moderate fibrosis and blood vessel proliferation, primarily of capillaries near the dermal-epidermal junction, and slight lymphoplasmacytic perivasculitis. The vascular proliferation within the fibrotic tissue was perpendicular to the orientation of the fibroblasts and to the epidermis. Moderate sweat gland dilation, the absence of hair in the hair follicles, and mild pigment incontinence were also observed. Masson’s trichrome staining confirmed collagen deposition in the dermis.

## Conclusions

Cicatricial retraction of the ear is a very frequent finding in cattle that recover from photosensitization (PS). It appears that, in cases of both PS and bee stings, fibrin present in the subcutaneous edema induces interstitial fibrosis with retraction of the myofibroblasts, which, in the ears, results in retraction and curving.

One of the primary clinical aspects considered in the present study was the occurrence of cicatricial retraction of the ears, a very common injury in cattle recovering from photosensitization (PS). The evidence indicates that both in non-fatal cases, in which there is subcutaneous edema induced by multiple bee stings on the head of cattle, and in cases without PS sequelae, fibrin present in the subcutaneous edema induces fibroblast proliferation and interstitial fibrosis; subsequently, due to the effect of myofibroblast contraction, there is bending of the pinna (a very common condition associated with PS, popularly known in Brazil as “cartridge-shaped ears”, or “*orelha encartuchada*”, in Portuguese).

The microscopic findings observed in the skin of the cattle in the present study were similar to those found in the skin of horses with chronic PS lesions caused by *Brachiaria humidicola*[19]. Therefore, chronic PS should be included in the differential diagnosis in animals with chronic skin lesions on the head that have survived multiple bee stings.

To distinguish PS lesions from lesions caused by bee stings, it should be considered that lesions caused by PS primarily affect the areas of the body with less pigmentation and with less hair coverage; in black and white cattle, alterations are concentrated in the white areas, while in Zebu breeds, the most severe lesions occur in areas with thinner skin (back of the thigh, ears, face, and udder). In addition, when animals with PS are exposed to sunlight, they show discomfort (sometimes intense), seek shade, and exhibit marked pruritus. Irritation and pain can be pronounced, with notable behavioral alterations, which can include aggressiveness; these signs are not reported in animals attacked by bees [20]. Additionally, in cases of hepatogenous PS, hepatic lesions can cause secondary conditions, such as icterus, bilirubinemia, bilirubinuria, as well as the presence of phylloerythrin in the serum and urine [20].

To clarify that the lesions reported in the present manuscript were not caused by hepatogenous PS secondary to ingestion of *Brachiaria* sp., we emphasize that these incidents occurred during the drought period, the animals did not exhibit icterus, and two of the affected animals were adults that did not exhibit previous lesions (PS due to ingestion of *Brachiaria* primarily affects cattle under two years of age). The calf of one of the cows affected did not become sick; additionally, of the 62 animals that were in the pasture, only the three that were attacked by the bees exhibited these lesions.

Prophylactic measures include keeping the animals away from areas with a history of bee accidents or pastures where there are beehives in trees or termite mounds; alternatively, beehives can be removed with the help of a beekeeper. It is recommended that inactive termite mounds be removed from grazing areas when possible because they can house not only bees but also other poisonous animals, such as snakes and scorpions.

## Ethics committee approval

The present study was approved by the Ethics Committee on Animal Use (CEUA) of the State University of Norte Fluminense (UENF), Rio de Janeiro, RJ, Brazil.

## Competing interests

The authors declare that they have no competing interests.

## Authors’ contributions

SAC wrote the first draft of the manuscript, gathered clinical and epidemiological data. FASG contributed to writing and analysis of clinical and epidemiological information. JSMB contributed to writing and evaluation of clinical aspects. MFR contributed to writing and epidemiological analysis. TP contributed to writing, editing clinical and epidemiological data, and histopathological analysis. PVP contributed to editing the manuscript and histopathological analysis. All authors’ read and approve the final manuscript.
